# Epidemiology and public health response in early phase of COVID-19 pandemic, Veneto Region, Italy, 21 February to 2 April 2020

**DOI:** 10.2807/1560-7917.ES.2020.25.47.2000548

**Published:** 2020-11-26

**Authors:** Francesca Russo, Gisella Pitter, Filippo Da Re, Michele Tonon, Francesco Avossa, Stefania Bellio, Ugo Fedeli, Lorenzo Gubian, Daniele Monetti, Mario Saia, Francesca Zanella, Manuel Zorzi, Elena Narne, Domenico Mantoan

**Affiliations:** 1 Regional Directorate of Prevention, Food Safety, Veterinary Public Health, Regione del Veneto, Padova, Italy; 2 Screening and Health Impact Assessment Unit, Azienda Zero, Regione del Veneto, Padova, Italy; 3 These authors contributed equally; 4 Regional Epidemiological Service Unit, Azienda Zero, Regione del Veneto, Padova, Italy; 5 Hygiene and Public Health Unit, Department of Cardiovascular Medicine and Public Health, University of Padova, Padova, Italy; 6 Informative Systems Unit, Azienda Zero, Regione del Veneto, Padova, Italy; 7 Clinical Governance Unit, Azienda Zero, Regione del Veneto, Padova, Italy; 8 Director General, Health and Social Area, Regione del Veneto, Padova, Italy

**Keywords:** SARS-CoV-2, COVID-19, Italy, coronavirus, pandemic

## Abstract

**Background:**

Veneto was one of the Italian regions hit hardest by the early phase of the coronavirus disease (COVID-19) pandemic.

**Aim:**

This paper describes the public health response and epidemiology of severe acute respiratory syndrome coronavirus 2 (SARS-CoV-2) infections in the Veneto Region from 21 February to 2 April 2020.

**Methods:**

Information on the public health response was collected from regional health authorities’ official sources. Epidemiological data were extracted from a web-based regional surveillance system. The epidemic curve was represented by date of testing. Characteristics of hospitalised COVID-19 cases were described and compared to those never admitted to hospital. Age- and sex-stratified case-fatality ratios (CFRs) were calculated.

**Results:**

Key elements of the regional public health response were thorough case-finding and contact tracing, home care for non-severe cases, creation of dedicated COVID-19 healthcare facilities and activation of sub-intensive care units for non-invasive ventilation. As at 2 April 2020, 91,345 individuals were tested for SARS-CoV-2 and 10,457 (11.4%) were positive. Testing and attack rates were 18.6 per 1,000 and 213.2 per 100,000 population, respectively. The epidemic peaked around 20 to 24 March, with case numbers declining thereafter. Hospitalised cases (n = 3,623; 34.6%) were older and more frequently male compared with never-hospitalised cases. The CFR was 5.6% overall, and was higher among males and people > 60 years of age.

**Conclusion:**

In the Veneto Region, the strict social distancing measures imposed by the Italian government were supported by thorough case finding and contact tracing, as well as well-defined roles for different levels of care.

## Introduction

At the end of 2019, China reported a cluster of cases of pneumonia of unknown aetiology in the city of Wuhan, Hubei Province [1]. A novel coronavirus was later identified as the causative agent [2] and called severe acute respiratory syndrome coronavirus 2 (SARS-CoV-2). In the following weeks, SARS-CoV-2 spread throughout China and around the world. The illness caused by SARS-CoV-2, denominated coronavirus disease (COVID-19), was declared a pandemic by the World Health Organization (WHO) on 11 March 2020 [3].

On 20 and 21 February 2020, the first two locally acquired cases of COVID-19 were reported in two northern Italian regions, Lombardy and Veneto [4,5]. The two cases were not linked with each other. Case 1 of the Veneto Region was a person in their 70s who had never travelled to China nor had any epidemiological link with China or a confirmed case. The case was admitted to a local hospital on 16 February with a severe respiratory insufficiency, tested positive for SARS-CoV-2 on 21 February and died on the same day.

Until 21 February, Italy had registered only three imported COVID-19 cases from China [4]. In a few weeks, more and more cases were discovered and the healthcare systems of the most affected regions were under enormous pressure [6]. According to the Government of Italy, as of 2 April 2020, a total of 119,827 COVID-19 cases had been identified across all Italian regions [7]. Veneto was the region hit fourth hardest, after Lombardy, Emilia-Romagna and Piedmont [7].

The objectives of this paper are to describe: (i) the Veneto Region’s early public health response from the start of the outbreak and (ii) the epidemiology of SARS-CoV-2 infections in the Veneto Region as at 2 April 2020.

## Methods

### Setting

The Italian healthcare system is universal, publicly funded and provides free care to all people. Every region is responsible for organising the healthcare system in its territory. Primary, community and hospital care are provided by local health units (LHUs). Each LHU includes a Department of Prevention, which is responsible for all prevention and public health activities, such as immunisation campaigns, infection prevention and control in the community, population-based screening programs, health promotion and environmental public health. The Veneto Region is located in north-eastern Italy, has a population of 4,905,854, and is one of the richest and most industrialised Italian regions [8]. Veneto’s regional healthcare system comprises nine LHUs and is characterised by a tight integration of hospital care, primary care and social services, and a strong central governance assured by the region through its technical body, Azienda Zero.

### Public health response coordination

The regional health authorities put a comprehensive public health response in place to contain the epidemic and to increase the capacity of the healthcare system. Already on 30 January, a regional task force coordinated by the regional Directorate of Prevention, Food Safety and Veterinary Public Health—composed of experts in public health, infectious diseases, virology, emergency medicine and intensive care medicine—was established to coordinate a response to the COVID-19 threat. After the emergence of the outbreak in the Veneto Region, a scientific committee was established to support the task force in the elaboration of protocols and guidelines.

### Data sources

In Italy, notification of infectious diseases is mandatory for all clinicians and public health professionals. An ad hoc, centralised information system was created by Azienda Zero to monitor the epidemic trend and track the pathways of COVID-19 cases in healthcare facilities, both for epidemiological and healthcare planning purposes. Data were gathered through a web-based application automatically fed by electronic reports of SARS-CoV-2 tests performed in all of the region’s laboratories. Clinicians and public health professionals can access this web-based application to retrieve laboratory results and to enter data on hospitalised COVID-19 cases, such as hospitalisation history and clinical conditions. Deaths that occurred among COVID-19 cases, both in hospitals and in the community, were registered manually by healthcare professionals and were periodically cross-checked with the regional population registry. Another web-based application was developed for contact tracing and management, and was linked to the previous one. This software allows the insertion of contact lists, the creation of links between cases and their contacts, and the management of all activities related to active surveillance and quarantine. Record linkage between the different applications is made through the regional population registry.

### Laboratory testing

Since January 2020, testing for SARS-CoV-2 was carried out on upper or lower respiratory samples by real-time RT-PCR targeting two different SARS-CoV-2 genes [9], according to WHO recommendations [10]. During the initial phase of the outbreak, up to 15 March 2020, the Regional Reference Laboratory at the University of Padova carried out all tests and samples were sent to the National Reference Laboratory at Istituto Superiore di Sanità (ISS) in Rome for confirmation. The test results were confirmed for all samples sent (n = 28). To face the increasing diagnostic demand, laboratory capacity was expanded by acquiring new instrumentation and authorising more laboratories to perform the test, under the supervision of the Regional Reference Laboratory. As at 2 April 2020, 14 laboratories across the Veneto Region performed real-time RT-PCR testing for SARS-CoV-2 [11].

### Definitions

Individuals were considered COVID-19 cases if their upper or lower respiratory samples tested positive for SARS-CoV-2 by real-time RT-PCR, regardless of the presence of symptoms.

Viral clearance of COVID-19 cases was defined as the presence of two consecutive negative SARS-CoV-2 tests performed at least 1 day apart [12].

COVID-19 cases were categorised into the following groups according to their healthcare pathway: (i) cases never admitted to the hospital during the course of illness, (ii) cases admitted to non-intensive care unit (ICU) wards during the course of illness and (iii) cases admitted to ICUs during the course of illness.

Case contacts were categorised as close contacts (those with high-risk exposure) and occasional contacts (those with low-risk exposure), according to the definitions provided by the European Centre for Disease Prevention and Control (ECDC) [13]. Briefly, high-risk exposure was defined as: having had face-to-face contact with a COVID-19 case within 2 m for more than 15 min; having had physical contact with a COVID-19 case; having unprotected, direct contact with infectious secretions of a COVID-19 case; sharing a closed environment with a COVID-19 case for more than 15 min or providing care to a COVID-19 case without using the recommended personal protective equipment (PPE). Low-risk exposure was defined as: having had face-to-face contact with a COVID-19 case within 2 m for less than 15 min; sharing a closed environment with a COVID-19 case for less than 15 min or providing care to a COVID-19 case while using the recommended PPE.

All deaths that occurred among cases were considered in this analysis, irrespective of their causal link with SARS-CoV-2 infection.

### Statistical analyses

The cumulative curve of COVID-19 cases and the curve of newly diagnosed daily cases were plotted using the date of testing as temporal reference. Daily numbers of new hospital admissions among COVID-19 cases, COVID-19 cases under home isolation and case contacts under quarantine were reported as well. We also reported the daily number of SARS-CoV-2 tests performed.

Descriptive statistics were run for the entire population of cases and stratified by type of healthcare pathway. Age-stratified attack rates and age- and sex-stratified case-fatality ratios (CFRs) were calculated. In a subset of hospitalised cases with complete information on the date of symptom onset (n = 2,199), we fitted a Weibull distribution to estimate the time-lag between the onset of symptoms and hospital admission.

Comparisons between strata were performed with the Mann–Whitney U test for continuous variables and with the chi-squared test for categorical variables. Differences were considered statistically significant at 0.05 α level (two-sided test).

Statistical analyses were performed using the software Stata version 14 (StataCorp, College Station, United States).

## Results

### Public health response to the COVID-19 epidemic

#### Social distancing measures

Case 1 lived in Vo’ Euganeo, a small town with roughly 3,300 inhabitants in the Province of Padova, and used to play cards at a local tavern. All regulars of the tavern underwent nasopharyngeal swabbing and most tested positive for SARS-CoV-2. In light of such findings, the regional government decided to offer SARS-CoV-2 testing to all residents of Vo’ Euganeo [5]. All positive cases (total n = 84) were isolated and their close contacts were quarantined.

**Figure 1:**
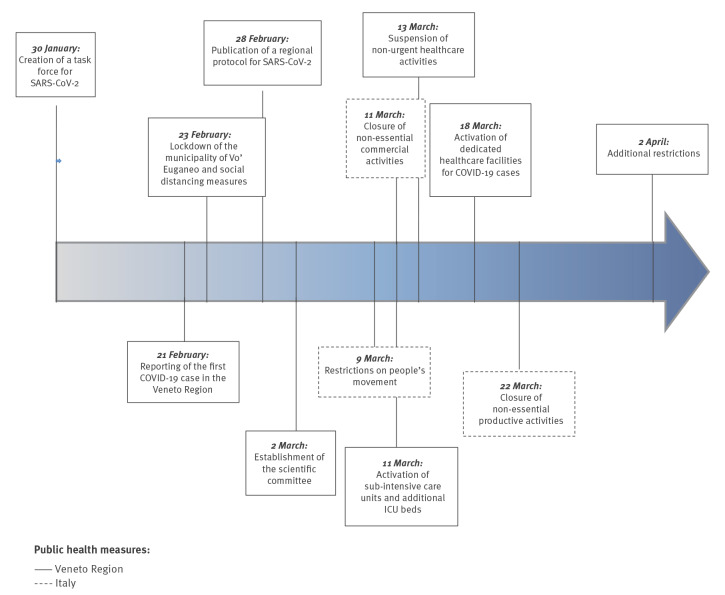
Timeline of public health measures adopted by the Italian and Veneto Region governments, Italy, January–2 April 2020

COVID-19: coronavirus disease; ICU: intensive care unit; SARS-CoV-2: severe acute respiratory syndrome coronavirus 2.

The timeline of public health measures adopted by the national and regional governments is depicted in Figure 1. On 23 February 2020, the Government of Italy issued a complete lockdown for 10 municipalities in the Lombardy Region and for Vo’ Euganeo in the Veneto Region. On the same day, the regional government, in agreement with the Ministry of Health, issued social distancing measures across the Veneto Region: schools and museums were closed, events and mass gatherings were banned and visitor access to healthcare facilities was limited. The Italian government imposed restrictions on people's movements across the country on 9 March, closure of non-essential commercial activities on 11 March and closure of non-essential productive activities on 22 March. The Veneto Region further strengthened these restrictions on 20 March by imposing a maximum distance of 200 m from home for walking dogs or necessary physical activities. A mass communication campaign at the national and regional levels—involving institutions, celebrities, companies and stakeholders—was conducted to inform all citizens about the need to stay home and follow all hygiene recommendations to protect themselves and the community from COVID-19.

#### Re-organisation of healthcare services

Healthcare services were re-organised to avoid any unnecessary patient visits to healthcare facilities and to devote most healthcare resources to the management of the epidemic (Figure 1). Since 13 March, non-urgent visits and surgical operations were suspended and patients were discouraged from visiting general practitioners and emergency departments. General practitioners were advised to limit office visits and replace them with phone assessments, followed by home visits when necessary. A detailed regional protocol was developed to manage individuals seeking medical attention for suspected COVID-19 in the most appropriate healthcare settings. The protocol dictated that patients with mild or no symptoms should stay at home in isolation and be managed by the primary and community care services of the LHUs.

Hospital admission was restricted to cases with severe symptoms such as high fever or difficulty breathing. A hotline was created to provide counselling to citizens and to carry out a preliminary assessment of suspected cases and case contacts, with subsequent referral to the appropriate level of care. In each hospital, separate pathways for access and care of individuals with suspected COVID-19 were instituted, and dedicated healthcare facilities were identified to admit COVID-19 cases. To reduce pressure on ICUs, ICU beds were almost doubled and sub-intensive care units were activated for non-invasive ventilation treatment of COVID-19 cases with respiratory failure. More details on the hospital system’s re-organisation has been described previously [14].

COVID-19 cases were discharged when clinically recovered, even if they still tested positive for SARS-CoV-2. After discharge, they were isolated at home or in dedicated healthcare facilities and were followed-up by territorial healthcare services until viral clearance was documented by real time RT-PCR. Detailed recommendations were given to all healthcare professionals and facilities on disinfection procedures, PPE and waste management.

#### Testing and contact tracing

The testing and contact tracing strategy progressively evolved during the epidemic. Until 21 February, according to national protocols, a suspected SARS-CoV-2 case was defined as a patient with both acute respiratory symptoms and a history of travel to China in the previous 14 days or an epidemiological link with China or with a confirmed case. Following the detection of the first locally acquired case, regional protocols were quickly updated to improve the sensitivity of the surveillance system and actions were taken by each LHU to improve contact tracing. Since 28 February, testing for SARS-CoV-2 was also recommended for any patient with severe acute respiratory infection or acute respiratory distress syndrome, as well as for patients with acute respiratory symptoms or influenza-like illness that were close contacts of a suspected, probable or confirmed case. Asymptomatic close contacts were traced and put under quarantine for 14 days since last contact with the case. Departments of Prevention assured daily active phone surveillance of asymptomatic cases and close contacts to monitor the development of symptoms. For close contacts, whenever any symptom compatible with COVID-19 appeared, the LHU arranged nasopharyngeal swabbing for SARS-CoV-2.

In addition to the social distancing dictated by the national government, the Veneto Region developed a comprehensive public health strategy on 17 March to prevent further spread of COVID-19. The main aim of this strategy was to stop all possible virus transmission chains by focusing on case finding, contact tracing and quarantining all possible case contacts (both close and occasional). Testing was extended to all case contacts, either symptomatic or asymptomatic. Each LHU’s Department of Prevention was strengthened with additional staff to pursue the strategy objectives. Moreover, systematic testing for SARS-CoV-2 was offered to all healthcare professionals (including nursing home personnel, general practitioners and pharmacists) and to essential services workers (policemen, firefighters, etc.), regardless of any known epidemiological link to a COVID-19 case.

### Epidemiology of SARS-CoV-2 infection in the Veneto Region

**Figure 2:**
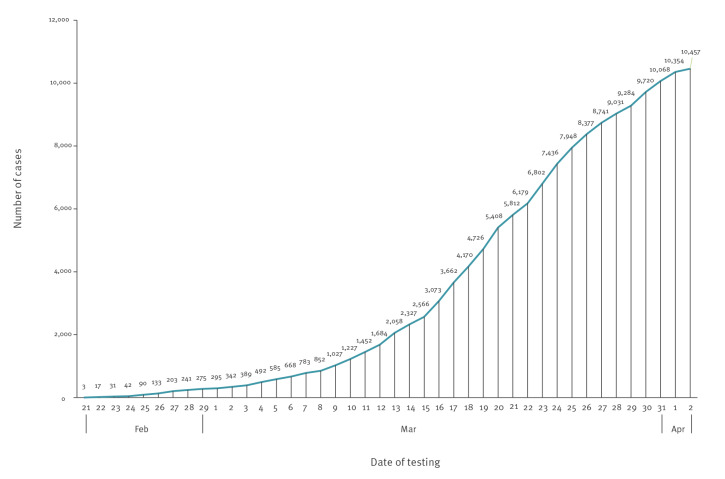
Cumulative number of COVID-19 cases, by date of testing, Veneto Region, Italy, 21 February–2 April 2020 (n = 10,457)

**Figure 3:**
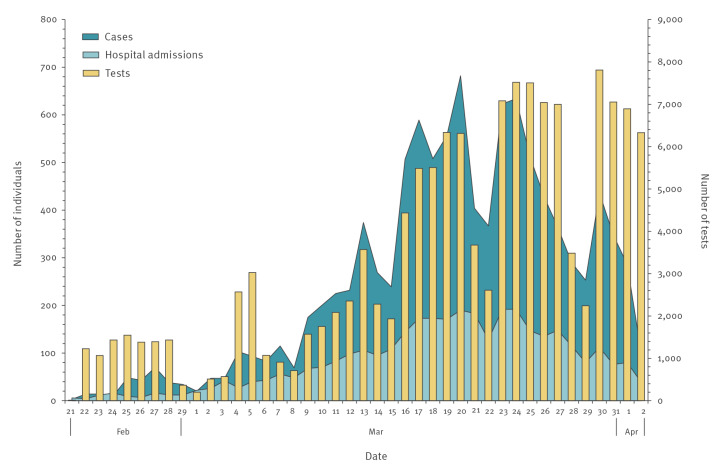
Daily number of SARS-CoV-2 tests, newly diagnosed COVID-19 cases and new COVID-19 case hospital admissions, Veneto Region, Italy, 21 February–2 April 2020

COVID-19: coronavirus disease; SARS-CoV-2: severe acute respiratory syndrome coronavirus 2.

**Figure 4:**
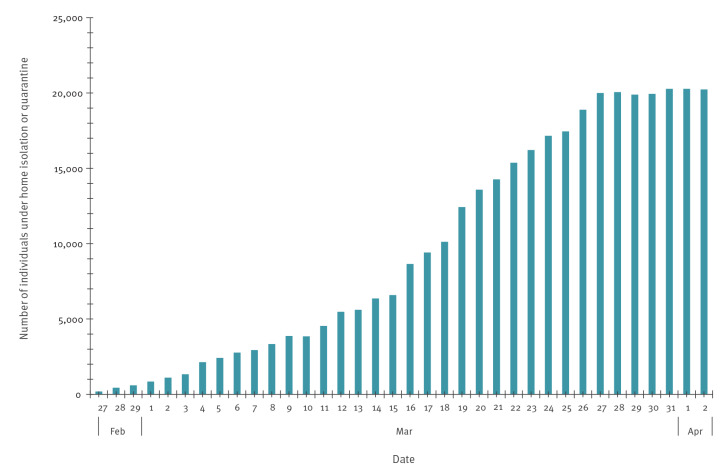
Number of COVID-19 cases under home isolation and case contacts under quarantine, by date, Veneto Region, Italy, 21 February–2 April 2020

Since the identification of the first case and up to 2 April 2020, a total of 91,345 individuals were tested for SARS-CoV-2, corresponding to a testing rate of 18.6 per 1,000 population. Of those tested, 10,457 (11.4%) individuals were positive, corresponding to an attack rate of 213.2 per 100,000 population. As shown in Figure 2, the cumulative number of COVID-19 cases increased steeply in the early phase of the epidemic and later levelled off. Figure 3 shows the daily number of performed SARS-CoV-2 tests, of newly diagnosed COVID-19 cases by date of testing and of new COVID-19 case hospital admissions by date of admission. Patients admitted to COVID-19–dedicated healthcare facilities in the community were not counted as hospitalised. The peak of both new diagnoses and hospital admissions was reached between 20 and 24 March. As depicted in Figure 4, the daily number of COVID-19 cases under home isolation and case contacts under quarantine increased up to roughly 20,000 on 27 March and remained steady thereafter.

COVID-19: coronavirus disease.

The number of newly diagnosed COVID-19 cases is reported by date of testing; the number of new hospital admissions is reported by date of admission.

COVID-19: novel coronavirus disease.

Some demographic and clinical characteristics of COVID-19 cases are shown in Table 1. Median age was 57.9 years (interquartile range: 29.6), 48.3% were male and 8.8% (n = 920) were healthcare workers. Since the beginning of the epidemic, viral clearance was documented for 784 of 10,457 cases (7.5%), while 581 (5.6%) died. Median age at death was 82.8 years.

**Table 1:** Demographic and clinical characteristics of COVID-19 cases, Veneto Region, Italy, 21 February–2 April 2020

Characteristics	All cases (n = 10,457)		Cases never admitted to the hospital (n = 6,834)		Cases admitted to non-ICU (n = 2,990)		Cases admitted to ICU (n = 633)	
	N	%	N	%	N	%	N	%
**Sex**								
Male	5,047	48.3	2,762	40.4	1,788	59.8	497	78.5
Female	5,410	51.7	4,072	59.6	1,202	40.2	136	21.5
**Age group (years)**								
0–9	92	0.9	86	1.3	6	0.2	0	0.0
10–19	170	1.6	161	2.4	9	0.3	0	0.0
20–29	731	7.0	687	10.1	37	1.2	7	1.1
30–39	905	8.7	836	12.2	60	2.0	9	1.4
40–49	1,494	14.3	1,250	18.3	206	6.9	38	6.0
50–59	2,240	21.4	1,624	23.8	490	16.4	126	19.9
60–69	1,511	14.5	772	11.3	564	18.9	175	27.7
70–79	1,327	12.7	464	6.8	649	21.7	214	33.8
≥ 80	1,987	19.0	954	14.0	969	32.4	64	10.1
**Outcomes**								
Infected healthcare workers	920	8.8	829	12.1	80	2.7	11	1.7
Viral clearance	784	7.5	428	6.3	307	10.3	49	7.7
Deaths	581	5.6	32	0.5	414	13.9	135	21.3

COVID-19: novel coronavirus disease; ICU: intensive care unit.

Table 2 shows the attack rates stratified for 10-year age groups. Attack rates progressively increased with age, with the lowest values found among the 0–9 years age group and the highest among the ≥ 80 years age group.

**Table 2:** Age-stratified attack rates of COVID-19, Veneto Region, Italy, 20 February–2 April 2020

Age group (years)	Number of COVID-19 cases	Attack rate (per 100,000 population)
0–9	92	22.2
10–19	170	36.1
20–29	731	151.5
30–39	905	165.4
40–49	1,494	194.6
50–59	2,240	283.9
60–69	1,511	253.9
70–79	1,327	269.6
≥ 80	1,987	572.4
**All ages**	**10,457**	**213.2**

COVID-19: coronavirus disease.

Overall, 34.6% (n = 3,623) of COVID-19 cases were admitted to the hospital; 28.6% were admitted to non-ICU wards and 6.1% to ICUs. Compared with cases never admitted to the hospital, hospitalised patients were more frequently male (63.1% vs 40.4%), had a higher median age (71.3 vs 52.4 years) and had a higher CFR (15.2% vs 0.5%). All of these differences were statistically significant (data not shown).

**Figure 5:**
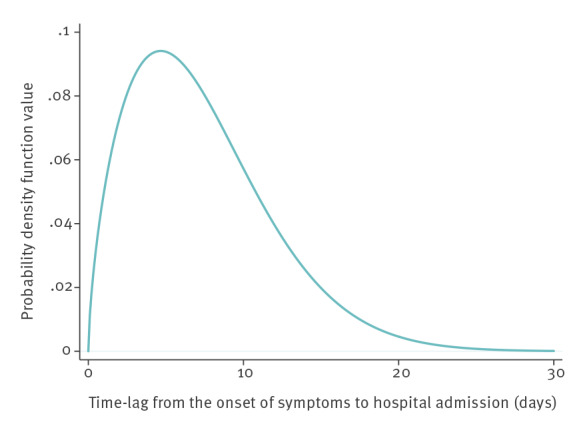
Weibull distribution of time-lag (days) from onset of symptoms to hospital admission among hospitalised COVID-19 cases, Veneto Region, Italy, 20 February–2 April 2020 (n = 2,199)

In the subset of 2,199 hospitalised patients with complete information on the date of symptom onset, the mean observed time-lag between the onset of symptoms and hospital admission was 6.9 days (SD 4.9 days). The Weibull distribution of time-lag is shown in Figure 5; the parameters of the Weibull distribution were as follows: β 1.65, η 8.19. CFR was strongly associated with age, ranging from 0.0% in the 0–39 years age group to 18.6% in the ≥ 80 years age group (Table 3). Above 50 years of age, the CFR was significantly higher in males than in females; overall, CFR was 7.2% in males and 4.0% in females.

COVID-19: coronavirus disease.

**Table 3:** Age- and sex-stratified deaths among COVID-19 cases and case-fatality ratio, Veneto Region, Italy, 20 February–2 April 2020

Age group (years)	All		Males		Females	
	Number of deaths	CFR (%)	Number of deaths	CFR (%)	Number of deaths	CFR (%)
0–9	0	0.0	0	0.0	0	0.0
10–19	0	0.0	0	0.0	0	0.0
20–29	0	0.0	0	0.0	0	0.0
30–39	0	0.0	0	0.0	0	0.0
40–49	1	0.1	1	0.2	0	0.0
50–59	21	0.9	13	1.2	8	0.7
60–69	40	2.6	34	3.7	6	1.0
70–79	150	11.3	116	14.6	34	6.4
≥ 80	369	18.6	200	26.1	169	13.8
**All ages**	**581**	**5.6**	**364**	**7.2**	**217**	**4.0**

CFR: case-fatality ratio; COVID-19: coronavirus disease.

As at 2 April, 1,064 hospitalised COVID-19 cases were discharged; among them, 24 were subsequently re-admitted to the hospital. The rate of repeated hospital admission was 0.2% among all cases and 2.3% among discharged cases.

## Discussion

The Veneto Region had one of the highest attack rates of SARS-CoV-2 infection during the early stages of the COVID-19 pandemic in Italy. Until 2 April, only Lombardy and Emilia-Romagna showed higher attack rates (457.9/100,000 and 330.6/100,000, respectively) [15]. From the beginning of the outbreak, the region chose a strategy of thorough contact tracing and case finding, and expanded this further during the course of the epidemic. This led to an increasing number of people—including COVID-19 cases with mild or no symptoms and close or occasional case contacts—put under home isolation or quarantine and active surveillance.

The number of newly diagnosed cases peaked at the end of March and then started to decline, paralleled by a declining trend of new hospital admissions. Of note, the epidemic curve was constructed by date of testing rather than by date of symptom onset, mainly because (i) date of symptom onset data were missing for many cases and (ii) date of symptom onset was not considered a good indicator of timing of infection, since COVID-19 cases may remain asymptomatic or develop symptoms several days after diagnosis.

Increasing evidence has shown that asymptomatic SARS-CoV-2 infection is common and that asymptomatic or pre-symptomatic cases can transmit the infection [16-24]. A modelling study in China estimated that 79% of all documented infections were attributable to undocumented cases [25]. Therefore, a wide testing policy, focusing not only on symptomatic individuals but also on asymptomatic case contacts, may be an essential component of the containment strategy [19,26,27]. In our view, the systematic search for cases and tracing of case contacts are of even greater importance after the severe social distancing measures have been relaxed, because prompt identification and isolation of new cases is crucial to prevent an uncontrolled resurgence of the epidemic [28-30]. To implement this strategy, the Veneto Region has developed a comprehensive, population-based data linkage approach and a real-time data analysis that considers all information on confirmed cases, case contacts, isolations, clinical conditions and active surveillance. During a massive epidemic, a public health strategy should also include a strong integration between primary care and the Department of Prevention in order to control pressure on the hospital network.

As at 2 April 2020, 34.6% of all COVID-19 cases were hospitalised, of whom 6.1% in an ICU. These figures differed from those of the Lombardy Region, where 47% of cases were admitted to hospital as at 8 March [31]. Such variance may reflect both different testing strategies, with Lombardy focusing more on symptomatic cases, and differently organised healthcare systems. In the Veneto Region, the network of primary and community healthcare and social services allowed the majority of patients without severe symptoms to be managed at home or in dedicated healthcare facilities in the community. Consequently, pressure on the hospital system in general and on ICUs in particular was kept under control even during the worst phase of the epidemic, as previously described [14]. This was also accomplished through the activation of sub-intensive care units, where patients with respiratory failure were treated with non-invasive ventilation. Such a multifaceted strategy prevented the saturation of ICUs, thus preserving the availability of high-quality intensive care for all patients that needed it, either COVID-19 or non-COVID-19.

Most patients requiring hospital admission were male and elderly individuals, in line with the available literature [27]. Overall CFR was 5.6%, higher than the overall CFR of 3.8% observed in China [32]. This difference is explained by the different age structures of the Veneto and Chinese populations, as confirmed by the very similar CFR observed in comparable age groups [4]. Of note, we observed a lower CFR than the 11.8% reported at the national level in Italy [15]. One possible explanation for this discrepancy may be the different testing strategies adopted by the regions. In particular, in Lombardy—the region that accounts for the majority of all Italian cases and deaths—testing was restricted mainly to severely symptomatic, hospitalised patients, which may have pushed the CFR upwards. Of note, follow-up was incomplete for some cases in this study; therefore, case-fatality figures should be read with caution. CFR was also significantly higher in males than in females, which may in part reflect a higher prevalence of comorbidities and of previous smoking among men.

Healthcare workers accounted for almost 9% of all cases, similar to the overall Italian proportion of 10% [15]. Since healthcare workers have a high chance of being tested for SARS-CoV-2, the relatively high proportion may be at least in part the consequence of increased detection in this particular population. However, healthcare workers are likely at higher risk of SARS-CoV-2 infection compared with the general population because of their occupational exposure. At the time of writing, we did not have not enough data to estimate the proportion of infections among healthcare workers that were acquired before risk-control measures were implemented, including the use of PPE. Future studies should focus on temporal trends of infections among healthcare workers to gain more insight on the effectiveness of these risk-control measures.

Strengths of this study are the large population under study and the reliability of the surveillance system based on automated electronic feeding of the dataset by laboratory reports. However, there are several limitations. The dataset had many missing data regarding information about clinical conditions of COVID-19 cases at diagnosis (asymptomatic/symptomatic, severity of symptoms, presence of comorbidities), the date of symptom onset and the type of exposure. Therefore, we were unable to reliably determine the proportion of asymptomatic cases, the epidemic curve by date of symptom onset, the proportion of cases linked to clusters, the most frequent exposure contexts (i.e. household, healthcare setting, etc.) and the serial interval. Another limitation is that we computed the CFR based on all the observed deaths among COVID-19 cases up to 2 April. As there is a time-lag of 2–3 weeks between onset of symptoms and death [33], we probably missed future deaths of cases recently infected and therefore may have underestimated the CFR. On the other hand, some cases may have died from other conditions not associated with SARS-CoV-2 infection, leading to an overestimation of the CFR.

In conclusion, the Veneto Region adopted a combination of severe social distancing measures, thorough case finding and contact tracing, and well-defined roles in different levels of care, with the aim of mitigating the effects of the COVID-19 pandemic and keeping pressure on hospitals under control. Formal evaluations of the impact of this set of interventions would be of utmost importance to inform public health and policy decisions in the future.
